# Nursery- vs. Mother-Reared Baboons: Reproductive Success and Health Parameters

**DOI:** 10.3390/vetsci11090416

**Published:** 2024-09-07

**Authors:** Sarah J. Neal, Steven J. Schapiro, Susan P. Lambeth, Elizabeth R. Magden

**Affiliations:** Department of Comparative Medicine, Michale E. Keeling Center for Comparative Medicine and Research (KCCMR), The University of Texas MD Anderson Cancer Center, 650 Cool Water Drive, Bastrop, TX 78602, USA; sschapir@mdanderson.org (S.J.S.); slambeth@mdanderson.org (S.P.L.); ermagden@mdanderson.org (E.R.M.)

**Keywords:** rearing, baboons, neutrophil-to-lymphocyte ratio, reproduction, wounding

## Abstract

**Simple Summary:**

Nursery rearing in nonhuman primates (NHPs), involving inanimate surrogates and/or human or conspecific peer rearing, is known to have negative consequences on behavior, immunology, and health, but it remains a necessary practice in cases of maternal rejection, neglect, abuse, injury, or illness. In baboons, there is mixed evidence of the effects of nursery rearing, including no differences between mother- and nursery-reared baboons in reproductive success and wounding but some differences in the prevalence of abnormal behavior. In the current study, we examined whether health parameters and reproductive success differ between mother- and nursery-reared baboons in a large captive colony. We found only minimal differences in health parameters, including a lower neutrophil-to-lymphocyte ratio (an indicator of inflammation, pathology, and stress in humans); body weight; and heart rate in nursery-reared baboons but no differences in reproductive success or wounding. These results may suggest that the collaboration between veterinary and behavioral management has yielded nursery-rearing protocols that result in healthy, robust baboons.

**Abstract:**

There is a plethora of data demonstrating the deleterious consequences of nursery rearing in nonhuman primates (NHPs). However, baboon studies report varying consequences of nursery rearing, from no differences in reproduction and sociality to moderate differences in social cognition and abnormal behavior. We compared health and reproductive parameters in a large sample (N= 231) of mother-reared (MR) and nursery-reared (NR) captive olive baboons housed at the Keeling Center for Comparative Medicine and Research, Texas. MR baboons had higher neutrophil-to-lymphocyte ratios and heart rates than NR baboons. Rearing was not a significant predictor of body condition score or body weight (*p* > 0.20), and MR and NR individuals did not differ in the level of wounding observed (*p* > 0.70). The proportion of successful births across NR and MR females was also not significantly different (*p* > 0.70), nor were rates of maternal neglect and infant death. These data suggest minimal differences in health and reproductive parameters across rearing statuses in baboons housed at this facility. In conjunction with previous research that also seems to show minimal differences as a function of rearing in baboons, but directly contrast with data in other NHP species, these data suggest that baboons may be more robust against deleterious effects of abnormal rearing conditions than other NHP species.

## 1. Introduction

In situations of maternal rejection, neglect, abuse, injury, or illness, captive nonhuman primates (NHPs) have historically been removed from their dams and reared in nursery settings [1]. Removing an infant from the dam can have a variety of negative consequences on infant development, ranging from altered immunological function to behavioral disturbances that can persist into adulthood [2,3,4]. Brent and Bode (2006) state that the “best way to produce healthy nonhuman primates that are able to interact socially, cope with environmental challenges, reproduce, and successfully rear offspring is to support a program of mother rearing.” Indeed, many studies in macaques have concluded that nursery rearing is the largest risk factor for the development of abnormal behaviors [4,5,6]. However, it is important to note that not all NHP species respond in the same way to nursery rearing [7]. Indeed, even members of the same genus (*Macaca* sp.) exhibit different patterns of behavioral responses to nursery rearing [8]. For example, whereas the presence of abnormal behaviors in nursery-reared rhesus macaques persist into adulthood, such abnormal behaviors seem to decrease or disappear with age in pigtail macaques (*Macaca nemestrina*) [9,10].

The types of housing, socialization, and enrichment greatly impact the success of rearing infant NHPs, and nursery-rearing practices have changed over time. Nursery-rearing practices and protocols were established and developed by veterinarians and behavioral managers in order to maximize the health and welfare of NHPs while minimizing adverse health and behavioral consequences. For example, the early hand rearing of NHPs focused on health assessments and clinical care, such as the formulas consumed, weight gains, medical issues, and maintaining a sanitary environment. However, this often resulted in a deficient social environment, and early nursery-rearing environments of social isolation caused severe behavioral abnormalities, along with immunological deficiencies, altered dopamine sensitivities, and altered brain structure developments [7,11,12]. The joint efforts between veterinary and behavioral management helped to spark improvements in the nursery environment, beginning with the inclusion of inanimate surrogates to which infants could cling, and further improvements were seen when those surrogated could swing/sway [13]. Peer rearing is another strategy to balance behavioral adaptability with necessary clinical care, with studies showing that the addition of early peer socialization may reduce the development of abnormalities reported in past studies that lacked conspecific interactions [7]. For example, a study that provided infant baboons with approximately one hour of peer socialization with 5–14 peers three times weekly found a reduction in abnormal behaviors when compared to single housing with human contact [1]. Furthermore, peer-reared nursery rhesus macaques showed no differences in affiliative or agonistic behaviors compared to mother-reared infants; however, they did exhibit an increase in non-injurious self-directed abnormal behavior, as well as a tendency toward increased anxiety-related behavior [14]. Lastly, surrogate–peer-reared rhesus macaques engaged in significantly more self-biting compared to peer-only and mother-reared infants [15] but have also been shown to exhibit lower cortisol in response to a stressor compared to mother- and peer-only-reared infants [16]. As such, there seems to be mixed evidence regarding the effects of peer-only, surrogate-only, and peer-surrogate rearing conditions.

There is a vast literature on the behavioral consequences of nursery rearing in NHPs, but there are immunological consequences as well. For example, nursery-reared (NR) rhesus macaques show higher lymphocyte proliferation in response to mitogen stimulation [17], have lower neutrophil counts, and seem to be less physiologically responsive (i.e., less cortisol increase in response to stressors) than mother-reared (MR) monkeys [2,16,18,19]. These studies suggest that nursery rearing alters hypothalamic–pituitary–adrenal (HPA) and immune function regulation [19,20]. Another measure of inflammation and stress, often used in human clinical medicine for its prognostic utility, is the neutrophil-to-lymphocyte ratio (NLR). This is calculated by dividing the number of neutrophils by the number of lymphocytes, and the normal range in humans is between 1 and 2. Values outside of this range (above 3 and below 0.7) are considered to be pathological indicators of inflammation, infection, cancer, psychiatric disorders, and, importantly, stress in humans [21,22,23]. Some evidence suggests that NLR may also indicate stress in NHPs. For example, across several studies, both macaques and baboons have been shown to exhibit a higher NLR following transport to a new facility, relocation to a new housing area, following restraint-chair training, and as a function of more sedations [21,24,25,26,27]. However, a *lower* NLR has also been associated with certain stressful circumstances in NHPs. Contrary to what we would expect given the stresses associated with nursery rearing in NHPs, both rhesus macaques and baboons show a lower NLR compared to MR individuals [21,27,28], and a lower NLR in infant rhesus macaques has been associated with higher stress values and emotionality, as well as a higher risk for deleterious health outcomes later in life [28]. As such, the data seem to suggest an association between NLR and stress in NHPs, but the type of stress may impact the direction of the effect [27]. Given the association with stress, NLR may have potential welfare utility with applications as a measure that can inform behavioral management practices and interventions [27].

While there is ample research on the effects of nursery rearing in macaques, fewer data exist regarding the effects of differential rearing in baboons. The data that do exist seem to demonstrate that only certain parameters differ between mother-reared (MR) and nursery-reared (NR) baboons and that some of these differences may diminish with age. For example, affiliative behavior in NR baboons was no different from that of MR individuals after 3 months of age, but aggression and abnormal behaviors remained higher in NR baboons well into adulthood [1]. This suggests that early differences in social behaviors may decrease upon housing in a more normal social environment, but the early environment may make nursery-reared baboons more susceptible to the development of stress-related and abnormal behaviors [29], a trait also observed in macaques [5]. Existing data also suggest some developmental differences, such as a lower body weight, a later and less intense adolescent growth spurt, and a later age of menarche onset in NR baboons [1]. Furthermore, it has been suggested that nursery rearing in baboons has consequences for health and longevity, as NR baboons tend to die at younger ages and have more incidences of diarrhea, although the lifetime incidence of wounding does not vary [1]. Regarding reproductive parameters, it seems that baboon infants raised in a nursery for the first two years of life exhibit no difference in live birth rates, pregnancy outcomes, or the ability to successfully raise infants to 180 days when compared to mother-reared infants [30]. It should be noted, however, that a majority of these behavioral, health, longevity, and reproductive data come from just two facilities of breeding baboons, and the data are up to 30 years old. As such (and given that nursery-rearing practices are continually refined), newer data from a different population of baboons are warranted.

While mother rearing of infants is considered the ideal rearing strategy, it is not without its risks and challenges in baboons. Indeed, even in situations of mother rearing, collaboration between veterinary and behavioral management is needed to minimize health risks and maximize the welfare and behavioral adaptability of infants. For example, in a large captive breeding colony of baboons, it was relatively common to observe dams displaying abusive behaviors towards their infants, which occurred in 55% of dams that had recently given birth [31]. Neglected and/or abused infants often require dam separation and nursery rearing, and as such, the need to provide nursery housing for infants, and for continued investigation into the best nursery practices, continues. Additionally, while previous data on NHPs show a variety of deleterious consequences of nursery rearing, there is mixed evidence of both the positive and negative consequences of peer rearing compared to surrogate–peer rearing, as well as seemingly minimal differences (but a paucity of data) in baboons. Therefore, we aimed to address some of the gaps in the literature regarding differences in health and reproductive parameters across mother- and nursery-reared baboons. We examined differences in parameters of health, including wounding, NLRs, body weights, body condition scores (a proxy for BMIs in NHPs [32]), heart rates, and respiration rates, as well as reproduction success, including rates of live birth and maternal neglect and abuse, as a function of rearing status.

## 2. Materials and Methods

### 2.1. Subjects

**Figure 1:**
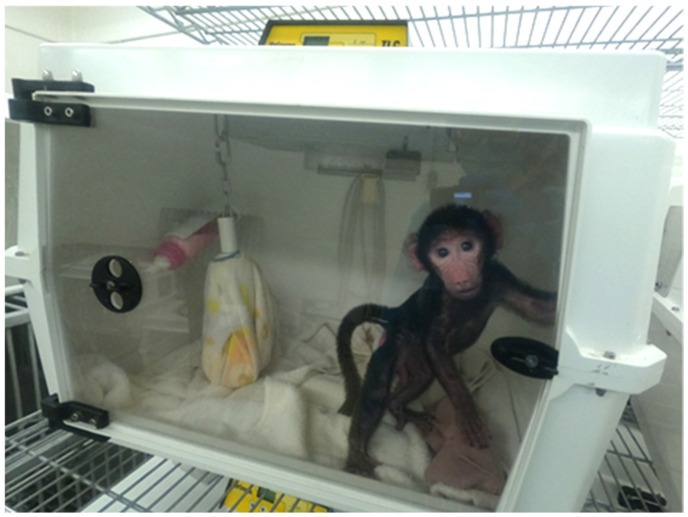
Infant baboon in nursery incubator with hanging surrogate and bottle.

The subjects were 231 baboons (*Papio anubis*) (165 females, 88 nursery-reared, Table 1) across 10 social groups included in this study. All baboons were housed in the Specific Pathogen-Free (SPF) breeding colony in either corrals or Primadomes™ with inside access or in indoor/outdoor runs. The SPF colony is a breeding research resource free of 18 pathogens and viruses that is maintained at The University of Texas MD Anderson Cancer Center, Michale E. Keeling Center for Comparative Medicine and Research (KCCMR), Bastrop, Texas. The baboons ranged in age from 0 to 21 years old (mean age = 5.66 years ± 4.56). Baboons were housed in breeding groups consisting of one or two breeding males, 12–16 breeding females, and their infant and juvenile offspring. As such, as is typical of other breeding colonies, there were significantly fewer adult males in our sample. Nursery-reared individuals were separated from their dam in the conventional baboon colony within 24 h following birth and were raised in a nursery incubator with a soft hanging cloth surrogate and human infant formula for the first three weeks of life (Figure 1). During week three, they began peer socialization for approximately 30 min/day and were started on wet biscuits and soft fruit. During week four, infants were moved out of the incubator and provided peer socialization twice daily with 3–4 peers. By week eight, infants spent all day in a play cage with same-age peer social groups. Infants were weaned during week twelve, at which time they resided, full time, in a play cage with their peers. They were moved to an outdoor housing facility with their peer group at one year of age. At two years of age, they were transferred to the larger SPF colony, if all bioexclusion agent tests were negative. Prior to moving to each next stage of housing, the baboons’ body weight, behavior (e.g., the presence of species-typical behaviors, including age-appropriate social behaviors and the lack of abnormal behaviors), and food intake were assessed to ensure each baboon was ready to progress to the next stage of the nursery. The baboons received a physical exam, hematology, and serum chemistry every six months while in the nursery and were monitored at least twice daily by care staff.

Mother-reared individuals were born to dams in the SPF colony and remained with their dams for at least the first 6 months of life.

Animals, when age-appropriate, had unlimited access to a high-fiber NHP diet (7195, Teklad, Inōtiv, West Lafayette, IN, USA) and water. In addition, they were fed fresh fruits and vegetables daily and were provided with regular enrichment items, such as forage, seeds, peanuts, raisins, peanut butter, and frozen juice cups. Subjects were also provided with destructible enrichment manipulanda and different travel or perching materials on a rotating basis to promote the occurrence of species-typical behaviors. Colony management practices included a comprehensive veterinary and behavioral management program to assess baboon health and psychological wellbeing along with daily environmental enrichment opportunities.

### 2.2. Data Collection

NLR values; the presence of an injury; body weights (kg); body condition scores (1–5, with 1 indicating emaciated, 3 indicating normal, and 5 indicating morbidly obese); heart rates (bpm collected manually); and respiration rates (RR: respirations per minute collected manually) were collected during physical exams in the fall of 2023. Blood samples for NLR were collected through a venipuncture of the femoral vein into EDTA anticoagulant tubes after the animals had been anesthetized with ketamine (10 mg/kg IM; Vedco, Saint Joseph, MO, USA). Blood sampling volumes were approved by the IACUC and a clinical veterinarian, and the baboons appeared healthy throughout the study. Blood samples were processed at the Keeling Center within 2 to 4 h of collection. To calculate NLR for each baboon, we divided the percent values for neutrophils by the percent values for lymphocytes, as we have done previously [21,27,35]. The age of the baboons at the time at which NLR values were obtained was used as the age variable.

During the physical exams, we recorded whether the baboons exhibited any type of injury upon examination. Descriptive notes were taken on the nature of the injury, and then these notes were coded to create an injury severity score, with 0 indicating no injury (*n* = 163); 1 indicating minor, old healing wounds (*n* = 45); 2 indicating wounding that required cleaning and topical Vetericyn^®^ (*n* = 19); and 3 indicating a wound that required analgesic or antibiotic medication (*n* = 4).

To examine reproductive success, we counted the number of live births, abortions, stillbirths, and in utero deaths for each female from 2017 (i.e., corresponding to their arrival on campus) to February 2024. We combined the number of abortions, stillbirths, and in utero deaths to create a measure of “unsuccessful births” and used the proportion of successful births as the dependent variable (the number of live births/total number of births = the proportion of successful births) to compare across rearing statuses (MR *n* = 53; NR *n* = 47).

To examine the rates of maternal neglect and cases of infant deaths within a group, we first counted the number of births in the SPF colony for each year (2021, 2022, 2023, and through June of 2024) and then counted the number of infants that were noted as being transferred to the nursery due to maternal neglect. Therefore, the rate of maternal neglect was equal to the number of neglected infants divided by the total number of births. We also counted the number of infants that were born (excluding stillbirths) that died before one year of age while still in the social group with the dam and noted the year in which the death occurred. Therefore, the rate of infant deaths prior to one year of age was equal to the number of infants that died divided by the total number of births within each year.

### 2.3. Data Analysis

Normality of the data was checked using histograms and QQ plots. All variables were relatively normally distributed except for NLR, which was positively skewed. Therefore, we used a Log10 transformation for NLR (hereafter referred to as “lg10NLR”), and we report the mean and standard error of the mean for both the raw and log-transformed data, where appropriate. A visual inspection of residuals and QQ plots of residuals by fitted values for log-transformed data showed good homoscedasticity. Additionally, because body weights differ between males and females, we calculated z-scores for the body weight for females and for males and used these body weight z-scores in analyses.

We used a multivariate ANCOVA with age as a covariate; sex and rearing as between-subject factors; and lg10NLR, body weight, BCS, HR, and respiration rate as dependent variables. To explore the possibility that rearing may affect these parameters at different ages, we selected only females in the dataset (due to the small number of males) and repeated the analysis with only juveniles, then with adults (5 years and older). We could not assess differences in infants or geriatrics due to the small number of NR individuals in those groups. We also could not assess interaction effects between sex and rearing given the small number of NR males.

We used a chi-square analysis to examine whether the numbers of different types of injuries (none, minor, moderate, or severe) differed across rearing statuses. Lastly, to examine the effects of rearing on reproductive success, we used a univariate ANOVA with the proportion of successful births as the dependent variable and rearing and age group as the between-subject variables. We also descriptively report the numbers and percentages of maternal neglect as well as infant deaths prior to one year of age. We used SPSS statistical software version 26 for all analyses (IBM, Armonk, NY, USA, 2021). Data are available from the corresponding author upon reasonable request.

## 3. Results

Using the entire sample, the multivariate ANCOVA showed that age was a significant covariate for body weight, BCS, HR, and RR (*p* < 0.001). After controlling for age, NR baboons had lower neutrophil-to-lymphocyte ratios (lg10NLR) [F(1,224) = 5.23, *p* = 0.023]; weighed less [F(1,224) = 5.38, *p* = 0.021]; and had lower heart rates than MR baboons [F(1,224) = 17.05, *p* < 0.001] (Table 2). The BCS and respiration rates were not significantly different as a function of rearing (*p* > 0.30).

To examine whether rearing affected NLR, body weight, BCS, HR, and RR differently across age groups, we re-ran the multivariate ANCOVA with only females across the different age groups. In juveniles (1–4 years of age), lg10NLR remained significantly lower in NR females (mean = 0.54 ± 0.06) compared to MR females (mean = 0.77 ± 0.06) [F(1,57) = 6.85, *p* = 0.011] and HR remained lower in NR females (mean = −145.52 ± 4.40) compared to MR females (mean = 162.48 ± 4.40) [F(1,57) = 6.59, *p* = 0.013]. In the adults (5 years and older), lg10NLR was lower in NR females (mean = 0.62 ± 0.06) than in MR females (mean = 0.83 ± 0.05) [F(1,86) = 3.12, *p* = 0.005], and body weight was lower in NR (z-score mean = 0.534 ± 0.08) compared to MR female adults (z-score mean = 0.79 ± 0.07) [F(1,86) = 6.21, *p* = 0.015].

**Figure 2:**
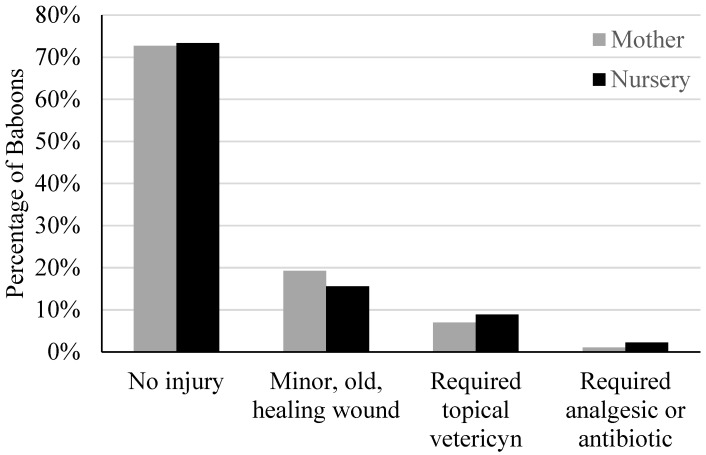
The percentage of MR and NR baboons showing different levels of wounding.

Regarding wounding prevalence across rearing statuses, MR and NR individuals did not differ in the level of wounding observed (*p* = 0.71). Figure 2 shows the percentage of baboons noted as exhibiting minor, moderate, severe, or no wounding.

Lastly, regarding reproductive success, there were no significant differences in the proportion of successful births between MR (0.962 ± 0.018) and NR females (0.951 ± 0.19; *p* = 0.71, *n* = 100). In total, 88% of the females in this study exhibited a 100% successful birth rate. Only 12 of the 100 females exhibited at least one unsuccessful birth between 2017 and June 2024, including 6 of 53 (11%) MR females and 6 of 47 (13%) NR females (Table 3). Regarding the rates of maternal neglect and infant death, the overall annual incidence of maternal neglect ranged from 0 to 5.9%, and the annual infant death ranged from 2.9 to 13.1%. A majority of the maternal neglect cases (60%) and cases of infant death prior to one year of age were from MR dams (68%, Table 4). Of note, one NR female rejected 3 infants, and one MR female rejected two infants over the course of this study period, together accounting for 50% of all maternal rejections included in this dataset. One MR female accounted for 3 of the 22 infant deaths.

## 4. Discussion

In sum, we found that NR baboons show a lower NLR, HR, and body weight and that the differences in NLR and body weight persist into adulthood. However, we found that wounding did not differ across MR and NR baboons, and reproductive success, including the proportion of live births and rates of maternal neglect and infant death prior to one year of age, did not differ between MR and NR females. Overall, in conjunction with previous data [1,17,29,30,34], these data suggest minimal differences in health, wounding, and reproductive parameters across rearing statuses in baboons.

One observed difference, consistent with previous studies in rhesus and chimpanzees [28,35], is that NLR was significantly higher in the MR compared to the NR baboons, and this difference was true in juveniles as well as adults. This is also consistent with previous data from our group showing a higher NLR in MR baboons [21]. As an indicator of stress, inflammation, and pathology in humans [23,36], this higher NLR may indicate that MR individuals experience a more stressful environment early in life compared to the less socially active nursery setting. Mother-reared infants experience all the social stress that is present in a breeding baboon population that grapples with dominance and low social rankings, preferred food guarding, fighting, and potential injuries [31,37,38]. Infants can become an easy target if one animal becomes aggressive with another, potentially resulting in infant injuries [39]. While the observation of these normal social interactions by baboon infants is beneficial for learning and social development, it is conceivable that there is increased stress, and thus, a higher NLR, associated with a close physical presence to the social conflict [21,27]. Indeed, the nursery environment eliminates some of this social stress, as well as other types of stressors present in large breeding groups, such as food and resource competitions, which may result in a lower NLR than baboons that experience those types of pressures. This also highlights that even under “ideal” conditions of mother rearing, a collaboration between veterinary and behavioral management is needed to minimize health risks in large breeding groups and to maximize the welfare and behavioral adaptability of infants.

Another possible explanation for the higher NLR in MR baboons is an unequal proliferation in the immune response cells. NR rhesus macaque infants had lower proportions of CD8 cells and natural killer cell activity and higher levels of lymphocyte proliferation in comparison to MR infants [17,40]. If nursery-reared animals exhibit higher lymphocyte values with no increase in neutrophils, this could cause a lower NLR, which is what we observed in the current study. Indeed, a cursory analysis of lymphocyte and neutrophil percentages across rearing in our sample shows that NR baboons have significantly higher lymphocyte but lower neutrophil percentages. Therefore, although this immune cell response difference between NR and MR monkeys was previously only reported in infants, it seems possible that the difference persists into adulthood, even after years of social, as well as outdoor, housing. This difference may reflect an altered maturation and/or physiological dysregulation as a result of perturbations during critical periods of development, causing an increased vulnerability to various diseases or pathological aging later in life [40].

We also found that NR baboons had a lower body weight and HR than MR baboons. Specifically, NR males had the lowest heart rates. Although this result should be interpreted with caution given the small number of NR males in our sample, this finding is not surprising, as it correlates with what is observed in human infants. Male newborn human infants have significantly lower baseline heart rates in comparison to females [41]. This sex-related heart rate difference was not observed in MR baboons, possibly due to their relative unfamiliarity with human interactions in comparison to NR infants. As such, similar to the increased cortisol observed in MR rhesus macaques following human handling [16], MR juvenile baboons may experience more sedation-related stress (and elevated heart rates) when they and their dams are sedated for physical exams. However, when analyses were limited to adult females (due to the low number of males in the sample), we found that heart rate no longer differed between MR and NR baboons. Conversely, body weight was only lower once females reached maturity, suggesting that differences across MR and NR in growth persist into adulthood, similar to differences in immune maturation, as discussed above. These results should be replicated with male baboons across age categories.

There were no significant differences in body condition scores, levels of wounding, or various measures of reproductive success across rearing statuses. The lack of significant differences in the parameters assessed, outside of NLR and body weight, likely indicates that the nursery strategy established by the KCCMR has been largely effective at raising healthy, reproductively normal baboons that are able to integrate into the larger established SPF baboon colony. However, we did not examine the reproductive success of males, an important feature for a breeding colony that warrants investigation. Additionally, we also only measured wounding at one point in time (during biannual physical exams in the fall of 2023) rather than wounding rates across time or over the lifespan. As such, future studies should examine wounding using a longitudinal approach. Lastly, we are still in the process of investigating whether NR and MR baboons show different rates of morbidity and mortality in our colony. Given that some of the differences found in the current study persisted into adulthood, it may be reasonable to expect that differences in other health parameters, including morbidity and mortality, may become more apparent with age. This highlights the need for additional data across a breadth of health parameters in MR and NR baboons.

There are many different nursery strategies discussed in the NHP literature, but most involve either surrogate–peer rearing (using both surrogate and peer socialization for varying time periods) or peer rearing only (socially housing infants with multiple peers and avoiding groups of only two animals, with most using groups of four or more infants). At the KCCMR, we implemented a rearing strategy similar to that implemented at the Health Sciences Center of the University of Oklahoma [42], which involved both the surrogate and peer rearing of infant baboons. Given that rearing practices can differ at different institutions, we are hesitant to make sweeping conclusions regarding the effects of nursery rearing on baboons. Overall, our data suggest some differences in health (i.e., NLR, body weight, and HR) but no differences in wounding and reproductive parameters in MR vs. NR baboons at our facility. Since most of these data are consistent with previous research showing similar differences (or a lack thereof), we suggest that baboons show minimal differences in health and reproduction parameters examined in this study as a function of rearing. It would seem that the nursery-rearing strategies and protocols established through the collaboration between veterinary and behavioral management, including the balance between clinical care and physical and behavioral health established over the years through empirical investigations of best (and worst) practices, result in healthy, robust baboons. Regardless, it would be beneficial to replicate these and other parameters, including behavior, morbidity, and mortality. One finding does seem clear: the existing baboon data directly contrasts with data in other NHP species that show vast deleterious effects of nursery rearing. This potentially suggests that baboons process or cope with nursery rearing differently than other NHP species, in that they seem to be somewhat resistant to the negative consequences of abnormal rearing conditions.

## Figures and Tables

**Table 1:** Demographics of the sample.

Age Group (years) [33,34]	Mother Reared		Nursery Reared		Total
	F	M	F	M	
Infant (<1)	12	13	2	0	27
Juvenile (1–4)	30	32	30	9	101
Adult (5–9)	32	3	26	4	65
Older Adult (10–14)	15	3	7	0	25
Geriatric (≥15)	3	0	8	2	13
Total	92	51	73	15	231

**Table 2:** Means and standard errors of outcome variables as a function of sex and rearing.

Dependent Variable	Mother Reared		Nursery Reared	
	F (*n* = 92)	M (*n* = 51)	F (*n* = 73)	M (*n* = 15)
lg10NLR *	0.773 ± 0.04	0.604 ± 0.05	0.585 ± 0.04	0.533 ± 0.09
Raw NLR	7.7 ± 0.54	5.33 ± 0.73	5.17 ± 0.49	4.37 ± 1.07
Body weight (z-score) *	−0.196 ± 0.07	0.26 ± 0.10	−0.09 ± 0.08	0.664 ± 0.17
BCS	2.94 ± 0.04	2.99 ± 0.06	2.96 ± 0.05	2.90 ± 0.10
HR (bpm) *	152.60 ± 2.51	150.16 ± 3.50	142.46 ± 2.86	126.78 ± 6.16
RR	46.11 ± 1.20	46.50 ± 1.67	45.57 ± 1.36	43.45 ± 2.95

Note: the asterisk (*) indicates a significant difference between mother-reared and nursery-reared baboons (*p* < 0.05).

**Table 3:** Dams with less than 100% successful birth rates.

Name	Rearing	Date of Birth	Age (years)	Successful Births (%)
Ju	MR	29 January 2018	5	50%
Za	MR	11 August 2014	9	50%
Tr	MR	23 November 2016	7	50%
Sa	MR	11 August 2016	7	67%
Mis	MR	29 November 2009	14	75%
Ya	MR	15 October 2008	15	88%
	**MR**	**Average**	**9.5**	**63%**
Be	NR	25 April 2018	5	50%
Tr	NR	5 June 2017	6	50%
Al	NR	15 March 2016	7	50%
Cj	NR	6 September 2007	16	67%
Mia	NR	28 February 2003	20	86%
Me	NR	29 March 2007	16	88%
	**NR**	**Average**	**11.67**	**65%**

**Table 4:** Cases of maternal neglect and infant deaths by rearing and year.

Year (# births)	Neglect		Total Neglect (%)	Death in Group		Total Death (%)
	MR	NR		MR	NR	
2024 (35)	0	0	0%	1	1	5.71%
2023 (93)	2	1	3.23%	5	2	7.53%
2022 (84)	2	1	3.57%	7	4	13.10%
2021 (68)	2	2	5.88%	2	0	2.94%
Total (% of total)	6 (60%)	4 (40%)	3%	15 (68.18%)	7 (31.82%)	7.32%

## Data Availability

Data are available from the corresponding author upon reasonable request.
